# Retrospective study of cisplatin plus radiotherapy toxicities in locally advanced squamous cell carcinoma of the head and neck – ReCisTT study

**DOI:** 10.3389/fonc.2024.1220640

**Published:** 2024-10-14

**Authors:** Ana Varges Gomes, Gilberto Castro, Thiago Bueno de Oliveira, Ana Medina Colmenero, Leonor Ribeiro, Amanda Psyrri, Nicolas Magné, Maria Plana Serrahima, Joana Marinho, Raul Giglio, Leticia Iglesias Rey, Martín Angel, Ana M. Macedo

**Affiliations:** ^1^ Medical Oncology Department, Centro Hospitalar Universitário do Algarve, Faro, Portugal; ^2^ Department of Medical Oncology, Instituto do Câncer do Estado de São Paulo, São Paulo, Brazil; ^3^ Medical Oncology Department, AC Camargo Cancer Center, São Paulo, Brazil; ^4^ Department of Medical Oncology, Fundación Centro Oncológico de Galicia, A Coruña, Spain; ^5^ Medical Oncology Department, Centro Hospitalar Universitário Lisboa Norte – Hospital Santa Maria, Lisbon, Portugal; ^6^ Medical Oncology Department, Attikon General University Hospital of Athens, Athens, Greece; ^7^ Department of Internal Medicine, National and Kapodistrian University of Athens, Athens, Greece; ^8^ Institut Bergonié, Department of Radiation Oncology, Bordeaux, France; ^9^ Institut Lucien Neuwirth, Department of Radiotherapy, Saint Priest en Jarez, France; ^10^ Oncología Médica, Institut Català d’Oncologia-Hospitalet, Instituto de Investigación Biomédica de Bellvitge (IDIBELL), Barcelona, Spain; ^11^ Medical Oncology Department, Centro Hospitalar Vila Nova de Gaia/Espinho, Vila Nova de Gaia, Portugal; ^12^ Associação de Investigação de Cuidados de Suporte em Oncologia (AICSO), Vila Nova de Gaia, Portugal; ^13^ Instituto de Oncología Ángel H. (AH) Roffo, Universidad de Buenos Aires, Buenos Aires, Argentina; ^14^ Department of Medical Oncology, Complejo Hospitalario Universitario de Ourense, Ourense, Spain; ^15^ Department of Genitourinary Cancer, Instituto Alexander Fleming, Buenos Aires, Argentina; ^16^ Department of Biomedical Sciences and Medicine Evidenze, Lisbon, Portugal; ^17^ Faculdade de Medicina e Ciências Biomédicas, Universidade do Algarve, Faro, Portugal

**Keywords:** SCCHN, concurrent chemotherapy, cisplatin, compliance, radiotherapy

## Abstract

**Introduction:**

Squamous cell carcinoma of the head and neck (SCCHN) is a multifactorial disease involving genetic and environmental factors representing one of the most frequent cancer-related deaths worldwide. Tobacco and alcohol use account for most SCCHN, while a growing subset of oropharyngeal cancers is causally associated with human papillomavirus (HPV) infection. Despite improvements in overall survival, patients with HPV-negative locally advanced (LA) SCCHN continue to have a poor prognosis. For these patients, the standard of care is radiotherapy with concurrent chemotherapy (RCT).

**Methods:**

This retrospective, multicenter, and observational study analyzed the treatment compliance of 326 patients with LA-SCCHN who underwent RCT between January 1st, 2014, and June 30th, 2017. This study also evaluated the potential factors associated with treatment compliance, the compliance impact on clinical response, and the main toxicities experienced by patients.

**Results:**

A total of 274 (84%) patients were compliant and received the planned dose of cisplatin. Overall, 957 adverse events were reported in 98.2% of patients during the study. The overall response rate was 80.2%, with 60.4% of patients achieving a complete response.

**Discussion:**

Despite the high treatment compliance, 62.6% of adverse events reported were related to cisplatin. Identifying risk factors associated with non-compliance could enable physicians to identify ineligible patients for cisplatin-based RCT and prevent patients from receiving inadequate treatment leading to severe adverse events..

## 1 Introduction

Squamous cell carcinoma of the head and neck (SCCHN) is one of the most frequent causes of cancer-related deaths, with more than 500,000 cases diagnosed annually (1). SCCHN is typically observed in the oropharynx, oral cavity, hypopharynx, or larynx, being a complex disease influenced by genetic and environmental factors (2). Although alcohol and smoking represent the major etiological risk factors, human papillomavirus (HPV) and poor oral hygiene also play a relevant role (3, 4).

Patients with locally advanced (LA) SCCHN (stage III or IVA/B) represent approximately 60% of the SCCHN cases at diagnosis (5). Despite improvements in overall survival due to therapy innovation, patients with HPV-negative LA-SCCHN continue to have poor prognoses (6). Thus, concurrent cisplatin-based chemotherapy (CRT) has been used with improved outcomes (7–9).

CRT with high-dose cisplatin is the standard of care for LA-SCCHN. Bolus cisplatin 100 mg/m^2^ x 3 cycles Q21 days concomitant with radiotherapy (RT) represents the standard of care for LA-SCCHN. The efficacy of cisplatin-based radiochemotherapy (RCT) seems to be correlated with the cumulative dose received (10, 11). Still, compliance with platinum-based RCT treatment might be lower due to the significant toxicity caused by this regimen (12, 13).

HPV-positive cancers are typically associated with better outcomes compared to HPV-negative tumors (14). Hence, the clinical benefit of cisplatin-based RCT has been shown to decrease with lower total dosage in patients with HPV-negative LA-SCCHN. Notably, a cumulative cisplatin dose of <200 mg/m^2^ has been associated with lower overall survival than a cumulative dose of ≥200 mg/m^2^ (15).

This retrospective observational study assessed compliance with cisplatin-based RCT in real-world patients with LA-SCCHN. This study also evaluated the potential factors associated with treatment compliance, the compliance impact on clinical response, and the main toxicities.

## 2 Methodology

### 2.1 Study design

This retrospective, multicenter, and observational study analyzed the treatment compliance in patients with LA-SCCHN treated with cisplatin-based RCT, administered between January 1^st^, 2014, and June 30^th^, 2017. All RCT regimens were allowed if the planned cumulative dose of cisplatin was defined before treatment as ≥200 mg/m^2^ body surface area (BSA). The study was conducted in 11 centers, including Portugal, France, Spain, Greece, Brazil, and Argentina.

### 2.2 Patients

Adults with histologically confirmed LA-SCCHN (stage III, IVA, or IVB according to the American Joint Committee on Cancer (AJCC) staging 7^th^ edition) of the oral cavity, oro- or hypopharynx, and larynx were enrolled in the study. Patients who had undergone surgery before cisplatin-based RCT, or were included in any clinical trial within 30 days before the RCT, were excluded.

### 2.3 Assessment

Medical history, prescriptions, and laboratory reports were collected from the patient’s medical records. The compliance was assessed based on the cumulative total dose of cisplatin ≥200 mg/m^2^ BSA. Patients receiving a cumulative dose of cisplatin <200 mg/m^2^ BSA were considered non-compliant.

The identification of a prognostic/predictive score for therapy compliance included the analysis of demographic characteristics (age, gender, body mass index, BSA, smoking/drinking status), disease characteristics (Tumor Nodes Metastases (TNM) classification, according to AJCC staging 7^th^ edition, disease stage, HPV/p16 status), medical history (previous malignant disease, Eastern Cooperative Oncology Group performance status (ECOG PS) at baseline, time since initial diagnosis until the start of cisplatin/RT, creatinine clearance [(140 – age) x mass (kg) x [0.85 if female]/72 x [serum creatinine (mg/dL)], and involuntary weight loss ≥20%).

Treatment objective responses were evaluated according to RECIST 1.1 (16). Adverse events (AEs) were also described according to National Cancer Institute-Common Terminology Criteria for Adverse Events (NCI-CTCAE; version 4.0).

### 2.4 Statistical analysis

Continuous variables are described as means, standard deviations (SD), or medians (interquartile range (IQR) and minimum and maximum) for variables with skewed distributions. Categorical and ordinal variables are described as absolute and relative frequencies. Compliance with cisplatin is presented as the number and percentage of patients who received a dosage of ≥200 mg/m^2^ cisplatin. A 95% confidence interval (CI) was calculated. A multiple logistic regression analysis was conducted to investigate the impact of explanatory variables on cisplatin compliance and to obtain a predictive score. The factors considered for the model included: demographic characteristics (age, gender, body mass index, BSA), disease characteristics (TNM classification, disease stage, HPV/p16 status), medical history (smoking/drinking status, ECOG PS at baseline, time since initial diagnosis until the start of cisplatin/RT, previous malignant disease, creatinine clearance, and involuntary weight loss ≥20%). A p-value of 0.05 significance level was considered. The Statistical Package for the Social Sciences (SPSS Inc., Chicago, IL, USA) software (version 22.0) was used to conduct the study analyses.

## 3 Results

### 3.1 Patients characterization

**Figure 1:**
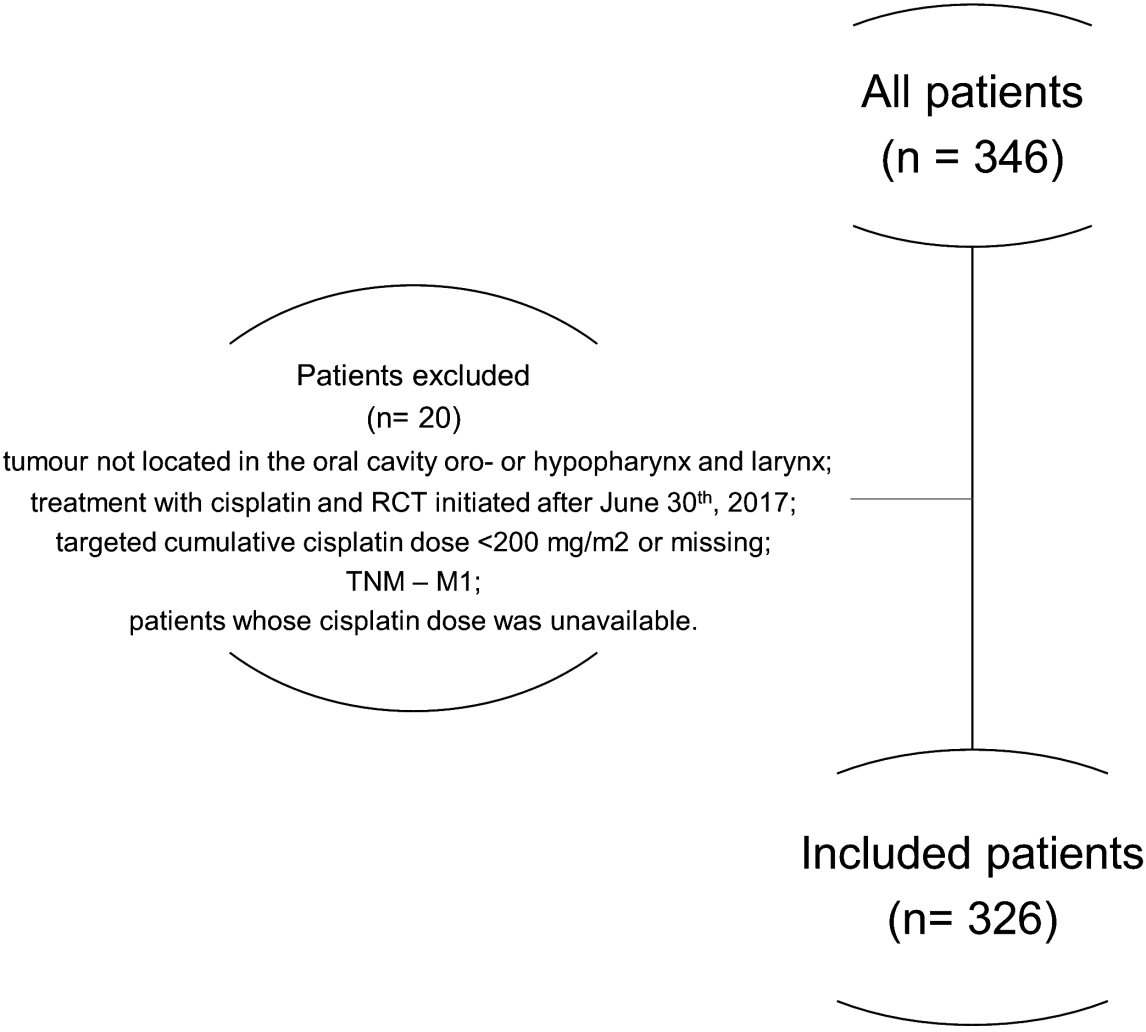
Description of the patient selection process.

A total of 346 patients were enrolled in the study (**Figure 1**). Twenty patients were excluded for not fulfilling the screening criteria: tumor location other than the oral cavity, oro- or hypopharynx, and larynx; treatment with cisplatin and RCT initiated after June 30^th^, 2017; targeted cumulative cisplatin dose <200 mg/m^2^ or missing; TNM – M1; or patients whose cisplatin dose was not available. Therefore, the final eligible population comprised 326 patients.

The demographic and clinical characteristics of the patients are summarized in **Table 1**. The median age was 63.0 years (SD± 8.9) and 86.2% were male. Overall, 5.2% of patients had prior malignancies, and less than 1.8% had previous renal dysfunction. Nearly half of the patients were smokers (48.5%), and 55.8% of patients consumed alcohol at least five times per week. Among patients who performed HPV testing (16.3%) and whose data were available (92.5%), 63.3% tested positive. Of the 24.5% of patients tested for p16, 48.8% were positive (**Supplementary Table 1**). The most common tumor location was the oropharynx (44.8%), followed by the larynx (21.8%) and hypopharynx (20.2%). Globally, 30.4% of the patients enrolled were LA-SCCHN stage III, 56.4% stage IVA, and the remaining were IVB (13.2%). The mean time from the initial diagnosis to the initiation of cisplatin-based RCT was 11.9 weeks. Most patients (92.3%) had an ECOG PS of 0 or 1 at the time of RCT initiation. Regarding renal function, the mean serum creatinine was 0.8 mg/dL, and the mean creatinine clearance was 90.1%. Hearing function status was not evaluated in most patients (87%).

**Table 1:** Baseline and clinical characteristics of the patients.

Variable		N= 326
**Gender, *n* (%)**	*Male*	281 (86.2)
	*Female*	45 (13.8)
**Age (years), median (IQR)**		63.0 (56.0-69.0)
**Body mass index, mean (SD)**		23.9 (4.9)
**Body surface area, mean (SD)**		1.7 (0.2)
**Prior malignancies, *n* (%)**		17 (5.2)
**Renal dysfunction, *n* (%)**		6 (1.8)
**Smoking status, *n* (%) ^(1)^**	*Non-smoker*	35 (10.7)
	*Smoker*	158 (48.5)
	*Former smoker*	131 (40.2)
**Alcohol consumption, *n* (%) ^(2)^**	*None*	81 (25.2)
	*Once a week or less*	31 (9.7)
	*Twice a week*	30 (9.3)
	*≥5 times a week*	179 (55.8)
**Primary tumor localization, *n* (%) ^(3)^**	*Oropharynx*	146 (44.8)
	*Larynx*	71 (21.8)
	*Hypopharynx*	66 (20.2)
	*Oral cavity*	62 (19.0)
	*Lymph nodes*	31 (9.5)
**HPV test performed, *n* (%)** HPV positive, *n* (%)		53 (16.3) 31 (63.3)
**P16 test performed, *n* (%)** P16 positive, *n* (%)		80 (24.5) 48 (48.8)
**LA-SCCHN stage, *n* (%)**	*III*	99 (30.4)
	*IVA*	184 (56.4)
	*IVB*	43 (13.2)
**ECOG PS, *n* (%)**	*0*	121 (37.1)
	*1*	180 (55.2)
	*2*	25 (7.7)
**Time since diagnosis to RCT initiation (weeks)**		11.9 ± 12.4

ECOG PS, Eastern Cooperative Oncology Group Performance Status; HPV, human papillomavirus; IQR, interquartile range; LA-SCCHN, locally advanced squamous cell carcinoma of the head and neck; RT, radiochemotherapy; SD, standard deviation.

(1) Missing data: n=1; (2) Missing data: n=4; (3) More than one option was allowed.

### 3.2 Treatment compliance

The cisplatin regimens used were cisplatin 100 mg/m² q3w in 198 patients (60.7%), cisplatin 40 mg/m² q1w in 103 patients (31.6%) and cisplatin 4 mg/m² days 1-4 q1w in 25 patients (7.7%). The mean cumulative cisplatin dose received during RCT was 251 ± 98.3 mg/m^2^ (**Table 2**).

**Table 2:** Cisplatin and radiotherapy administration.

Cisplatin administration (N= 326)	
**Total dose received** (mg/m^2^) ^(1)^	
Mean ± SD	251 ± 98.3
Min-Max	30-600
**Treatment duration** (weeks)	
Mean ± SD	5.47 ± 6.14
Min-Max	0-10.43
**Reasons for treatment discontinuation**, n (%)	165 (50.6)
Adverse events	146 (44.8)
Progressive disease	3 (0.9)
Death	5 (1.5)
Loss-to-follow-up	2 (0.6)
Other	9 (2.8)
Not reported	161 (49.4)
Radiotherapy administration (N= 326)	
**Treatment duration** (weeks)	
Mean ± SD	6.5 ± 4.7
Min-Max	0-59
**Reasons for treatment discontinuation**, n (%) ^(2)^	14 (4.3)
Adverse events	7 (50.0)
Progressive disease	1 (7.1)
Death ^(3)^	5 (35.7)
Loss-to-follow-up	1 (7.1)

SD, standard deviation. (1) Total dose of received cisplatin; (2) Missing data: n=2; (3) 2 dealths were due to adverse events related to cisplatin administration, 2 were due to adverse events from radiotherapy, and 1 due to progressive disease.

Overall, 274 (84.0%, 95% CI: 79.7% - 87.6%) patients were compliant and received the planned dose of ≥200 mg/m² BSA. The mean treatment duration was 5.47 weeks. Cisplatin treatment was prematurely discontinued in 50.6% of patients, mainly due to AEs, in 146 (88.5%) patients. Regarding RT administration, the median duration was 6.5 weeks. Overall, 4.3% of patients discontinued RT because of AEs (**Table 2**).

The multivariate regression analysis showed that ECOG PS was the only factor independently associated with cisplatin treatment compliance during RCT (ECOG PS 2 HR: 0.135 [95% CI: 0.35 - 0.528]; p = 0.004), underlining that patients who had an ECOG PS of 2 were less likely to be compliant with cisplatin-based RCT (**Supplementary Table 2**).

**Figure 2:**
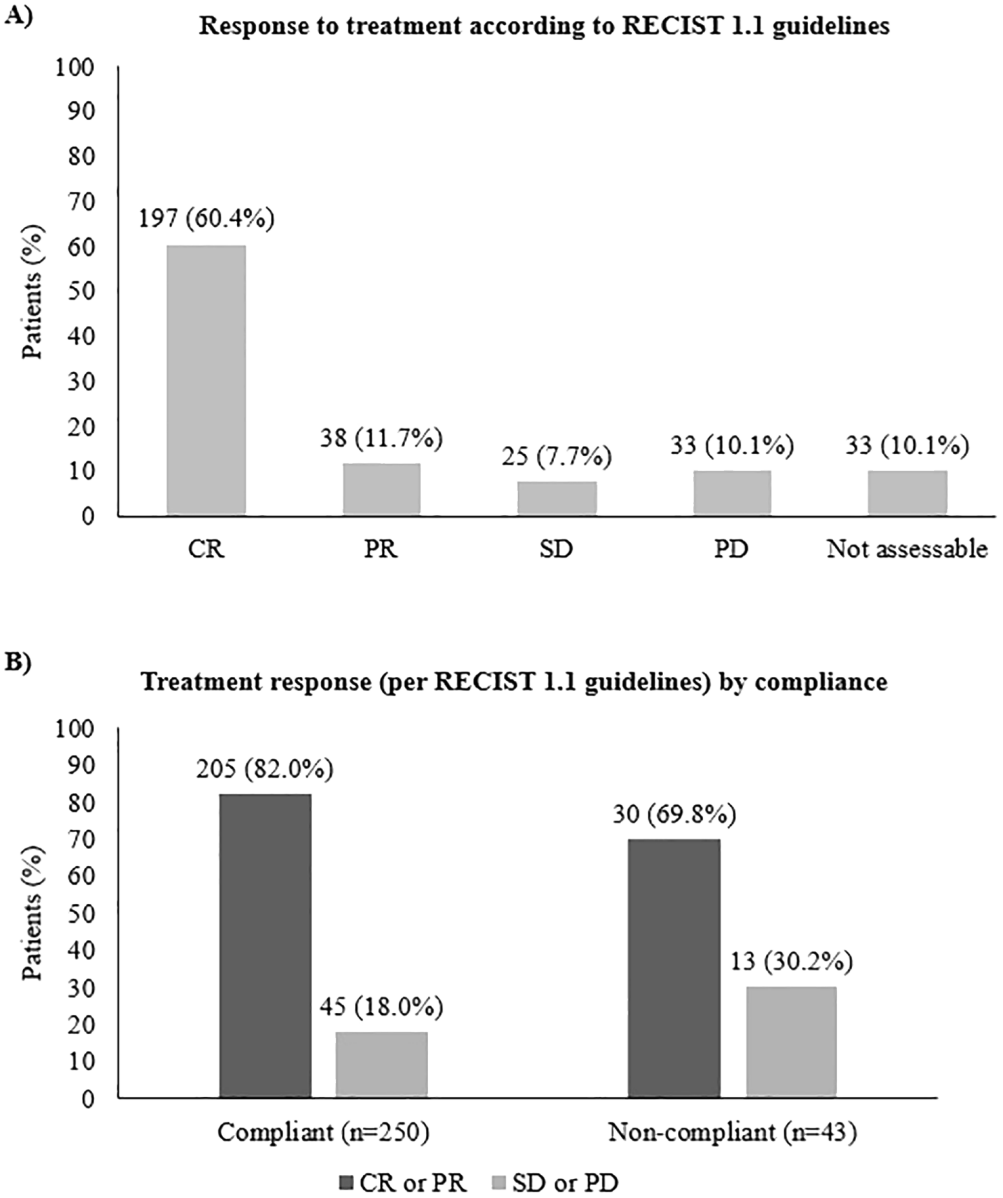
Response to cisplatin-based RCT **(A)** in the overall population and **(B)** according to compliance with treatment. CR, complete response; PR, partial response; SD, stable disease; PD, progressive disease.

When analyzing the radiological response to cisplatin-based RCT, the overall response rate (ORR) was 80.2%, with 60.4% achieving a complete response (**Figure 2A**). While an ORR of 82.0% was achieved in compliant patients who received a total dose of cisplatin ≥200 mg/m², in non-compliant patients (who received a total dose of cisplatin <200 mg/m²), the ORR was 69.8%. This difference was not statistically significant between groups (p-value = 0.063, **Figure 2B**), with an estimated odds ratio of 1.97 [95% CI: 0.9547 - 4.0817].

### 3.3 Safety analysis

A total of 957 AEs were reported in 98.2% (n= 320) patients during the study. Overall, 62.6% (n= 599) of AEs reported were related to cisplatin. Of the total AEs reported, 74.1% (n= 709) were mild to moderate (grade 1-2), 23.2% (n= 222) were grade 3, and 2.3% (n= 22) were grade 4. Severe adverse events (SAEs) were registered in 8.4% (n= 80), and 75.0% (n= 60) were related to cisplatin. The most common AEs related to cisplatin during RT were oral mucositis (26.6%, n= 159), nausea and vomiting (16.1%, n= 97), and neutropenia (7.7%, n= 46). Additionally, 43 (7.2%) AEs were related to impaired renal function. **Table 3** summarizes the AEs that registered more than 20 occurrences.

**Table 3:** Adverse events.

Adverse event	n (%)	Severity (CTCAE v4.0)			
		Grade 1-2	Grade 3	Grade 4	Grade 5
Total AEs	957	709 (74.1%)	222 (23.2%)	22 (2.3%)	4 (0.4%)^(1)^
AEs related to cisplatin	599 (62.6%)	427 (71.3%)	158 (26.4%)	12 (2.0%)	2 (0.3%)
Total SAEs	80 (8.4%)	–	–	–	–
SAEs related to cisplatin	60 (6.3%)	–	–	–	–
AEs related to cisplatin during RCT					
* Oral* *mucositis*	159 (26.6%)	96	60	3	–
* Nausea/* *vomiting*	97 (16.1%)	79	17	1	–
* Neutropenia*	46 (7.7%)	34	10	2	–
* Renal* *involvement*	43 (7.2%)	21	19	3	–
* Weight loss*	35 (5.8%)	25	10	–	–
* Anaemia*	28 (4.7%)	22	6	–	–
*Dermatitis* *radiation*	25 (4.2%)	25	–	–	–
* Asthenia*	21 (3.5%)	21	–	–	–

AE, adverse event; CTCAE v4.0, Common Terminology Criteria for Adverse Events, version 4.0; RCT, radiochemotherapy; SAE, severe adverse event.

(1) 2 deaths were due to AEs related to cisplatin treatment, 2 were due to AEs related to radiotherapy.

## 4 Discussion

This study revealed that patients with LA-SCCHN achieved a high compliance rate with cisplatin concomitantly administered with RT, which is adversely affected by poor ECOG PS. This study also disclosed that non-compliance might be associated with lower tumor response.

Though the clinical characteristics of these patients are heterogeneous, as they were collected in a real-world scenario, they are similar to populations studied in previous clinical trials. Regarding the TNM stage at diagnosis, most patients had T3 to T4 (78.2%) and were N2 (52.5%), respectively, similar to that of the GORTEC 2007-02 trial (14). Only 53 (16.3%) patients were tested for HPV and 80 (24.5%) for p16, values slightly lower than those previously published (17). The lower level of testing might be explained by the inclusion dates of the study, as HPV testing has only in recent years been recommended and implemented. Notably, it should be highlighted that performing HPV testing in oropharyngeal cancer patients is critical since HPV-positive cancers are commonly associated with better outcomes compared with HPV-negative tumors (14).

This international multicentered study disclosed a high treatment compliance rate (84%) among patients with LA-SCCHN. This compliance rate is notably higher than the one reported in a previous retrospective study (37%, COMPLY) conducted in patients with LA-SCCHN (stage III or IVA/B) who received a cumulative dose of ≥200 mg/m², based on their age, ECOG, and renal function (17). While the most common regimen used in our study was 100 mg/m² q3w, in the COMPLY study, the most common regimen was 40 mg/m² q1w, aiming to prevent toxicity (17). Of note, in the COMPLY study, the compliance rates were higher for lower-dose weekly schedules (77.8% for 40 mg/m^2^ and 65% for 30 mg/m^2^) than those obtained with a high-dose schedule (54.8% for 100 mg/m^2^). Still, in another retrospective study, nearly half of all patients completed three cycles of high-dose cisplatin (18).

The high-dose cisplatin regimen used in this study is considered the most adequate, as it promotes better locoregional and overall survival relative to RT following international clinical guidelines (19, 20). Still, it was associated with a higher incidence of AEs, as most toxicities are dose and schedule-dependent (17), which, in turn, might potentiate dose reductions and treatment delays with this intensive regime (12).

Identifying risk factors in patients who do not tolerate high cisplatin doses could enable physicians to identify ineligible patients for cisplatin-based RCT and prevent excessive toxicities and severe AEs (21). Accordingly, this study assessed the factors potentially affecting treatment compliance with cisplatin, such as demographic and clinical features and other SCCHN-related characteristics. The ECOG PS was the only factor identified by the multivariate model as an independent factor associated with compliance with cisplatin during RCT. Patients with an ECOG PS of 2 were less likely to comply with cisplatin-based RCT, suggesting that it should be considered a risk factor for compliance, and, thus, alternative treatments might be considered. These results are consistent with the international guidelines recommending that patients older than 70 years with an ECOG PS of ≥2 should not be treated with cisplatin (22, 23). Accordingly, most studies on CRT excluded patients older than 70 years. Nonetheless, in this model, age was considered a continuous variable; perhaps stratification of this variable could have revealed certain significant age ranges as predictors of compliance with cisplatin-based RCT. Another possible explanation is that as biological age *per se* is currently not recognized as a good predictor of benefit and/or toxicity in elderly patients, it should not guide treatment decisions and the functional age should be adopted instead (24). Decision-making in older patients requires a multidisciplinary evaluation and risk assessment provided by a comprehensive geriatric assessment.

The association between compliance and objective response to cisplatin-based RCT was also assessed. Suboptimal compliance with the cisplatin regimen might negatively impact patient outcomes. Regardless of the treatment regimen, it has been suggested that a cumulative dose of 200 mg/m² has to be achieved to allow a therapeutic benefit (12). A higher objective response was obtained in compliant patients (82.0%) who received a total dose of cisplatin ≥200 mg/m² compared with non-compliant patients (69.8%) who received a total dose of cisplatin <200 mg/m², with an estimated odds ratio of 1.974. These results suggest that the cumulative cisplatin dose might correlate with clinical response to cisplatin-based RCT.

The compliance to cisplatin plus RT is frequently lowered due to toxicity (17). Despite the high treatment compliance in this study, 62.6% (n= 599) of reported AEs were cisplatin-related. Most were low grade (grade 1 or 2) and aligned with those previously reported, including oral mucositis, neutropenia, and renal impairment (14). Strategies such as more frequent lower RT doses could potentially circumvent high-dose related toxicities, thereby improving compliance. A recent Japanese study found that weekly RCT was not inferior to 3-weekly RCT for post-operative high-risk patients with LA-SCCHN and had a favorable toxicity profile, leading the authors to conclude that this could be a new strategy in these patients (25, 26). Although there is lack of robust scientific evidence, weekly lower dose RCT may also benefit elderly patients who are frail (27). Other strategies such as accelerated fractionation with concomitant boost and RCT with concurrent split-dose cisplatin are also being explored as alternatives for patients who cannot safely tolerate high-dose RCT (28, 29). The main limitation of this study is its retrospective nature, as all the available data depends on the information already registered on the medical records. Still, this population represents the real-world setting, with a high prevalence of comorbidities and risk factors. Furthermore, this study was performed during the COVID pandemic, which impacted the recruitment period as the workload of clinicians/researchers increased considerably. Lastly, as HPV testing was not the diagnostic standard of SCCHN during the period the patients were diagnosed, we were unable to do subgroup analysis by HPV status due to the low number of patients with data available.

To our knowledge, this is the most extensive study that has provided up-to-date real-world data on compliance with cisplatin during RCT in patients with LA-SCCHN. The cisplatin regimen was administered according to each country’s site and local practices without being biased by screening criteria. Thus, the results from our retrospective study might be extrapolated to the general population of patients with LA-SCCHN receiving cisplatin.

This study shows a high cisplatin compliance rate (median cumulative dose ≥200 mg/m^2^) in patients with LA-SCCHN receiving cisplatin-based RCT, which is negatively influenced by ECOG PS. The absence of cisplatin compliance during RCT adversely affects tumor response.

## Data Availability

The original contributions presented in the study are included in the article/**Supplementary Material**. Further inquiries can be directed to the corresponding author.
