# Supplementary material for: Retrospective study of cisplatin plus radiotherapy toxicities in locally advanced squamous cell carcinoma of the head and neck – ReCisTT study
Source: Front Oncol. 2024 Oct 14;14:1220640. doi: 10.3389/fonc.2024.1220640 (PMC11513364; doi:10.3389/fonc.2024.1220640)
Supplement: Supplementary file 1 [file Table1.docx]

Supplementary Material

Retrospective study of cisplatin plus radiotherapy toxicities in locally advanced squamous cell carcinoma of the head and neck – ReCisTT Study

**Ana Varges Gomes^1,*^, Gilberto Castro Jr.^2^, Thiago Bueno de Oliveira^3^, Ana Medina Colmenero^4^, Leonor Ribeiro^5^, Amanda Psyrri^6,7^, Nicolas Magné^8,9^, Maria Plana Serrahima^10^, Joana Marinho^11,12^, Raul Giglio^13^, Leticia Iglesias Rey^14^, Martín Angel^15^, Ana M. Macedo^16,17^**

*** Correspondence:** Ana Varges Gomes, MD, [anafmferreira@yahoo.com](mailto:anafmferreira@yahoo.com)

# Supplementary Tables

**Supplementary Table 1**. **HPV test results.**

|  | **n** | **%** |
| --- | --- | --- |
| **HPV test (N=326)** |  |  |
| Performed | 53 | 16.3 |
| Not performed | 225 | 69.0 |
| Non applicable | 48 | 14.7 |
| **Results of HPV test (n=53)** |  |  |
| Positive | 31 | 58.5 |
| Negative | 18 | 34.0 |
| No information | 4 | 7.5 |
| **P16 test (n=326)** |  |  |
| Performed | 80 | 24.5 |
| Not performed | 246 | 75.5 |
| **Result of P16 test (n=80)** |  |  |
| Positive | 39 | 48.8 |
| Negative | 41 | 51.2 |

Supplementary Table 2 – Multivariable regression analysis of factors associated with compliance with cisplatin during RCT.

| **Variables** | **HR** | **Lower** | **Upper** | **p-value** |
| --- | --- | --- | --- | --- |
| Age (continuous variable) | .952 | .904 | 1.003 | .062 |
| Sex (reference category: female) | 1.224 | .381 | 3.929 | .734 |
| Smoking Status (reference category: smoker) |  |  |  | .224 |
| *Ex-smoker* | .584 | .259 | 1.319 | .196 |
| *Non-smoker* | .362 | .098 | 1.336 | .127 |
| Alcohol Intake during a week (reference category: none) |  |  |  | .877 |
| *Once a week or less* | 1.809 | .401 | 8.173 | .441 |
| *Twice a week* | 1.030 | .207 | 5.125 | .971 |
| *≥5 times a week* | 1.012 | .360 | 2.839 | .983 |
| Body Mass Index (continuous variable) | .972 | .895 | 1.055 | .497 |
| LA SCCHN stage (reference category: III) |  |  |  | .255 |
| *IVA* | .599 | .178 | 2.013 | .407 |
| *IVB* | 1.794 | .249 | 12.951 | .562 |
| ECOG PS before the start of RCT (reference category: 0) |  |  |  | .010 |
| *1* | .748 | .318 | 1.760 | .506 |
| *2* | .135 | .035 | .528 | .004 |
| Time between diagnosis and first treatment (weeks) | 1.044 | .994 | 1.098 | .088 |
| Previous malignant disease: no (reference category: yes) | .488 | .083 | 2.852 | .426 |
| Does the subject have any involuntary weight loss ≥20% since the initial diagnosis? no (reference category: yes) | 1.247 | .336 | 4.626 | .741 |
| Creatinine clearance rate (continuous variable) | 1.007 | .989 | 1.024 | .456 |
| HPV test performed |  |  |  | .476 |
| *Negative* | .198 | .022 | 1.756 | .146 |
| *Not performed* | .338 | .057 | 1.998 | .232 |
| *Not available* | .270 | .042 | 1.739 | .168 |
| TNM (T) classification |  |  |  | .995 |
| *T1a* | .587 | .000 | . | 1.000 |
| *T1b* | 2.471 | .000 | . | 1.000 |
| *T2* | .000 | .000 | . | .999 |
| *T3* | .000 | .000 | . | .999 |
| *T4a* | .000 | .000 | . | .999 |
| *T4b* | .000 | .000 | . | .999 |
| TNM (N) classification |  |  |  | .622 |
| *N1* | 1.029 | .288 | 3.684 | .965 |
| *N2a* | 1.576 | .292 | 8.510 | .597 |
| *N2b* | .488 | .143 | 1.665 | .252 |
| *N2c* | .484 | .147 | 1.593 | .233 |
| *N3a* | .475 | .060 | 3.792 | .483 |
| *N3b* | .301 | .011 | 8.633 | .484 |
| ECOG Eastern Cooperative Oncology Group; HPV: human papillomavirus; LA SCCHN: Locally advanced squamous cell carcinoma of head and neck; PS: performance status; RCT: Radiochemotherapy; TNM: Tumor Node Metastasis. | | | | |
